# Species Richness and Host Associations of Lepidoptera-Attacking Tachinidae in the Northeast Ecuadorian Andes

**DOI:** 10.1673/031.009.3901

**Published:** 2009-06-02

**Authors:** John O. Stireman, Harold F. Greeney, Lee A. Dyer

**Affiliations:** ^1^ Department of Biological Sciences, 235A, BH, Wright State University, 3640 Colonel Glenn Highway, Dayton, OH 45435; ^2^ Yanayacu Biological Station & Center for Creative Studies, Cosanga, Ecuador, c/o 721 Foch y Amazonas, Quito, Ecuador; ^3^ Biology Department, University of Nevada; Reno, Nevada

**Keywords:** parasitoid, Diptera, biodiversity, caterpillars, tropical rainforest, morpho-species, insect rearing

## Abstract

Most of the unknown biological diversity of macro-organisms remaining to be discovered and described lies in the tropical regions of the world and consists primarily of insects. Those insects with parasitoid lifestyles constitute a significant portion of insect diversity, yet parasitoids are among the most poorly known of major insect guilds in the humid tropics. Here we describe and analyze the richness of one diverse taxon of parasitoids, flies in the family Tachinidae, reared from Lepidoptera as part of a biological survey of Lepidoptera and their parasitoids in one mid-elevation (2000 m) area in the northeast Ecuadorian Andes. One hundred fifty-seven separable tachinid “morpho-species” were reared from approximately 160 species of Lepidoptera in 16 families. These tachinid flies were recovered from a sample of over 12,800 successful caterpillar rearing events that resulted in either adult Lepidoptera or parasitoids. Tachinid species accumulation and rarefaction curves exhibit no sign of reaching an asymptote and richness estimators indicate that the community likely consists of nearly twice this number of species (at minimum). Most tachinid species were reared infrequently, with 50% being represented by a single individual. The majority of species appeared to be relatively specialized on one or a few related hosts, but sampling was insufficient to make strong inferences regarding host range. The tribes Blondeliini and Goniini were the best represented, but some tribes that were expected to be common such as Tachinini and Winthemiini were poorly represented. The estimates of tachinid species richness derived here are suggestive of a far more diverse tachinid community than in temperate localities in North America. Additional rearing of Lepidoptera, as well as other herbivorous insect taxa, along with the use of additional collecting methods will be necessary to achieve a more accurate understanding of the richness of tropical Tachinidae and their contribution to broader patterns of tropical biodiversity.

## Introduction

Despite more than a quarter millennium of exploration, categorization and description of organismal diversity, our understanding of the number of living species on earth is relatively poor (Gaston and Spicer 2004). This failure to gain a comprehensive understanding of diversity is due in part to growing use of molecular tools to explore biodiversity, which has opened a window into the hitherto unappreciated magnitude of microbial species richness that we are only beginning to understand (e.g., Rappé and Giovannoni 2003). However, estimates of the diversity of macroscopic organisms, such as insects, are nearly as variable and weakly supported as those of micro-organisms, with estimates of global insect species numbers ranging from as few as 4.8 million (Odegaard 2000) to over 30 million (including other arthropods; Erwin 1982). Much of the uncertainty in estimates of insect diversity is due to our poor understanding of tropical biodiversity (Gaston 1991; Godfray et al. 1999). Although there has been considerable debate regarding the extent of the undescribed richness of tropical insects (e.g., Novotny et al. 2002), one conclusion that researchers tend to agree upon is that we desperately need more surveys and documentation of tropical insects and their ecological interactions (Godfray et al. 1999).

One guild of tropical insects for which our knowledge is perhaps poorest, and which has the greatest potential for influencing estimates of insect diversity upwards, is the insect parasitoids (e.g., Brown 2004). Our poor understanding of tropical (and temperate) parasitoid biodiversity is due in part to their frequently small size, specialized ecological niches, and relatively small population sizes associated with their elevated trophic positions. Godfray (1994) estimated that parasitoids represent 8.5% of described insect species, yet some workers suggest that the fraction of insect species that possess parasitoid lifestyles may exceed 20% (LaSalle and Gauld 1991). Thus, to achieve an accurate appreciation of global insect diversity, we need to obtain reliable estimates of the diversity of parasitoid lineages in the tropics.

**Figure 1.**
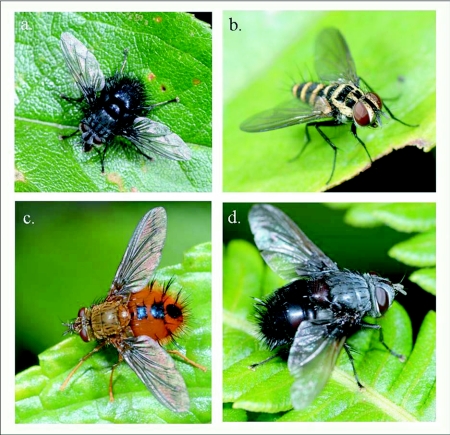
Examples of some of the Lepidoptera-attacking tachinid taxa commonly encountered around Yanayacu Biological Station. a. Goniini: *Gaediopsis* sp. b. Blondeliini: *Leptostylum* sp. (probably), c. Tachinini: *Epalpus* sp. d. Goniini: *Leschenaultia* sp. (photos by JOS).

The focus of the current study is to document the diversity (species richness) and host associations of one particular group of tropical parasitoids, flies in the family Tachinidae (Figure 1). The family Tachinidae contains approximately 9,200 (Brown 2001) to 10,000 (Irwin et al. 2003) described species and ranks second in species richness among families of Diptera. All species of Tachinidae for which life histories are known are parasitoids of other arthropods, primarily insects. As parasitoids, tachinid flies are second only to the vast “Parasitica” group of Hymenoptera in diversity and ecological importance (Godfray 1994; Stireman and Singer 2003a). They are particularly frequent and apparent as parasitoids of larval Lepidoptera, especially macrolepidopteran familes such as Arctiidae, Noctuidae, Sphingidae and Nymphalidae (Gentry and Dyer 2002, Stireman and Singer 2003a; Janzen and Hallwachs 2008). Although tachinid parasitoids tend to be larger in size and more conspicuous than most of their hymenopteran parasitoid counterparts, the species richness and host-use patterns of tropical tachinids is equally obscure. In fact, despite their diversity and ecological importance as natural enemies, there has been little quantitative exploration of tachinid diversity at local, regional, or global scales (though see Stireman and Singer 2003b; O'Hara 2007; Stireman 2008).

Current estimates of the species richness of Tachinidae among geographical provinces suggest that the Neotropical region harbors the largest number of species and represents a geographic epicenter of tachinid diversification (Stireman et al. 2006; O'Hara 2007). The Neotropical region boasts an estimated 2,864 described species (Guimarães 1971) belonging to an impressive 822 genera, almost twice as species rich as any other geographic realm (O'Hara 2007). Although tachinids are found in a wide variety of habitats in the tropics and from sea level to alpine tundra, their diversity is most apparent at middle elevations (1000–2000m) along the mountain chains of tropical Central and South America, where tachinids are an abundant and conspicuous component of the diurnal insect fauna. Despite the large number of described species, it is generally thought that only a fraction of Neotropical Tachinidae have been described, and for most of these nothing is known about their life histories, host associations, or behavior (Guimarães 1977).

Here, we present preliminary analyses of the species diversity and host affiliations of Tachinidae reared from lepidopteran larvae the northeast Ecuadorian Andes. We present a preliminary list of genera and species reared and provide host—family affiliations for most taxa as well as notes on the taxonomy of the species reared and associations with host genera. In addition, the taxonomic composition of the tachinid community and emergent patterns of host-associations and host specificity are examined.

## Materials and Methods

### Collection

**Figure 2.**
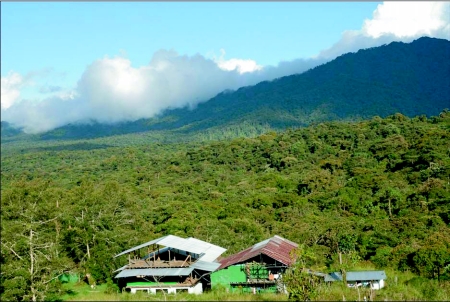
Yanayacu Biological Station and the surrounding “hanging valley” of montane cloud forest where caterpillar hosts of tachinids were collected and reared (Napo Province, Ecuador).

All tachinids in this study were reared as part of a collaborative biological survey and inventory project initiated in 2001 that is focused on surveying and inventorying plant-caterpillar-parasitoid associations in an Ecuadorian cloud forest (Stireman et al. 2005; Dyer et al. 2007). The survey project is centered at Yanayacu Biological Station & Center for Creative Studies (YBS), located at 2200m in the Quijos Valley, Napo Province, in northeastern Ecuadorian. Much of the ca. 2000 hectare reserve of adjacent Cabana San Isidro and YBS is relatively level cloud forest, some of the only remaining habitat of this type in the Andes (Figure 2). Although most collections of caterpillars were made within 1–3 km of the station, collections were also made at higher and lower elevation sites in the surrounding region, primarily within 20 km. Because boundaries between sampling areas are difficult to define and relatively few caterpillars were sampled in these outlying areas, all samples were considered together. Thus, the sampling area should be considered to be on the order of 200 km^2^ and encompass elevations from 500–3000 m (although only a small fraction of this area was sampled).

Caterpillars were systematically collected from 10m^2^ plots by Ecuadorian parataxonomists and Earthwatch volunteers. Vegetation within plots was visually scanned and all encountered caterpillars were collected along with material from their host plant. Additional caterpillars were collected opportunistically as they were encountered along trails and streams. Each individual caterpillar was assigned a unique number and the species (or morpho-species) of the caterpillar and its host-plant was recorded. Caterpillars were reared individually in clear plastic bags or glass jars in an open-walled, shaded rearing shed at ambient temperature and humidity. Every two days, bags were cleaned and foliage was replaced. After pupation, individuals were checked regularly and emerged adult Lepidoptera or parasitoids were collected and preserved. Throughout this process life history data were recorded (e.g., host, host plant, collection date, pupation date, eclosion date). Host plants were primarily identified by E. Narvaez (Universidad Central de Ecuador) and C. Chicaiza (Herbario Nacional Ecuatoriano). Caterpillars were assigned temporary morpho-species names if the species was unknown and later, if an adult was reared, added to the pool of specimens awaiting identification by taxonomic experts associated with the biological inventory project. Each caterpillar reared to either an adult moth/butterfly or a parasitoid is referred to as a “rearing event”.

Upon eclosion, tachinids were killed by freezing and subsequently pinned along with their puparia. Initially these collections were stored at YBS in boxes under ambient (indoor) conditions, which resulted in humidity-associated damage to many specimens. When reliable 24-hour electric power became available at YBS, pinned specimens were stored in a -20°C freezer. Accumulated specimens were then sent to the laboratory of JOS at Wright State University for sorting and identification.

### Identification

Tachinid specimens were initially sorted to genus using a key to Central American Tachinidae developed by D.M. Wood (Wood, in prep.), which is based, in part, on his generic key of Nearctic Tachinidae (Wood 1987). A number of specimens could not be assigned to genera on this basis for one of three reasons: 1) some reared specimens from Ecuador belong to genera not represented in the Central American fauna, 2) several specimens appeared to belong to undescribed genera (D.M. Wood pers. comm.), 3) some of the morphological distinctions between established genera appear to break down in the Neotropical fauna (e.g., see Wood 1985). Generic determinations for genera not present in Wood's unpublished key were determined in part by inspection by D.M. Wood, comparison with identified specimens in the Canadian National Collection of Insects (CNC) and/or with reference to C.H.T. Townsend's Manual of Myiology (1936–1945). These determinations should be treated as preliminary until further more focused taxonomic work is done with this material. JOS was unable to sort a small number of heavily damaged adult specimens to genus and these are omitted from this study.

After division into genera, specimens were further sorted into morpho-species with reference to external morphology (primarily chaetotaxy, wing patterns, shape and structure of head appendages, and color patterns). For some groups, further identification was made using keys and descriptions from the primary literature, through inspection by D.M. Wood or J.E. O'Hara, and by comparison with identified specimens in the CNC. For most specimens, however, specific determinations could not be made. Sorting of specimens into morpho-species was complicated by the fact that many “species” were represented by only one or a few individuals, and of those represented by multiple individuals, sometimes only a single sex was available for examination. Furthermore, it was often difficult to determine where intraspecific morphological variation ended and interspecific variation began, especially in several large genera in which there appear to be many undescribed species (e.g., *Siphona, Erythromelana, Lixophaga, Calolydella*). For this reason, estimates of the number of species and their host associations should be interpreted with some caution, and a low estimate (conservative “lumping” approach) of species richness is also provided. Although the divisions of individuals here are properly considered “morpho-species,” they are referred to as species for simplicity. All reared and sorted specimens are currently housed in the Wright State University Collection of Insects. As further identifications are made and confirmed, voucher specimens will be returned to Ecuador and be housed in the Museo Ecuatoriana de Ciencias Naturales in Quito, Ecuador.

Relatively few of the host caterpillars from which tachinids have been reared have been identified to species at this time and have been given temporary morphospecies designations. Thus, the exact number of host species is uncertain. Many host species are currently being identified by collaborators on this Biological Survey and Inventory survey of caterpillars and parasitoids in the Ecuadorian Andes.

### Analysis

Observed and estimated (rarefaction) species accumulation curves were calculated and plotted using the software EstimateS 7.5 (Colwell 2005). The rarefaction curve was calculated using the Mao Tau estimator (Colwell et al. 2004) with 1000 randomizations. EstimateS was also used to estimate the following species richness estimators and their 95% confidence limits (based on 1000 randomizations without replacement): ACE, ICE, Chao-1, Chao-2, Jack-1, Jack-2, Bootstrap and Michaelis-Menton (see Chao 2005, Colwell 2005, and references therein for detailed descriptions of these estimators). The accumulation of singletons and doubletons over time was also plotted with this software. A least-squares regression was used to assess the relationship between host range and the number of rearings for each tachinid species using PAST 1.8 (Hammer et al. 2001). Significance was assessed with 10,000 permutations. PAST 1.8 was also used to assess which commonly used model (log-series, log-normal, geometric, or broken stick) best fits the observed species abundance distribution.

## Results

Four hundred eighty-nine adults representing approximately 157 species and 50 genera of Tachinidae were reared from 357 rearing events of approximately 160 morpho-species of Lepidoptera in 16 families (Table 1). In addition, one sarcophagid species (*Boettcheria* spp.) was reared on two occasions from larvae of an unknown saturniid (Table 1). This estimate of species number represents the number of morphologically differentiable groups of individuals and must be treated with some caution. A secondary examination of the specimens in which individuals deviating only slightly in morphology were “lumped” together results in a more conservative estimate of 139 species. An appreciable number of additional tachinid parasitism events were recorded in which the adult tachinids failed to eclose from observed puparia.

Considering all data generated by the caterpillar rearing project, the overall parasitism frequency (including all parasitoid taxa) is approximately 11% (based on >28,000 caterpillar records). Of the parasitoids identified to family (whether larvae, pupae, or adults), 37% were Tachinidae, with the remainder consisting of Hymenoptera, primarily Ichneumonoidea. Many more tachinids, as well as other parasitoid taxa, likely perished in hosts that succumbed to pathogens or other sources of mortality (e.g., less than 50% of collected Lepidoptera resulted in an adult insect, whether it be moth or parasitoid). If only caterpillar samples that produced an adult moth/butterfly or parasitoid are considered, the estimate of the total parasitism frequency rises to 24.8% (3186 of 12805 records).

**Figure 3.**
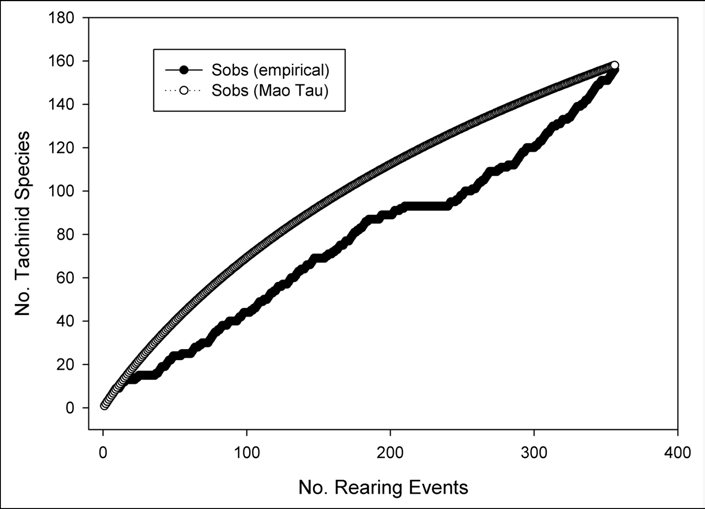
Accumulation curves of tachinids species against the number of rearing events. Sobs (empirical) equals the observed number of species, Sobs (Mau Tau) is the mean rarefied Mau Tau estimate of observed species based on 1000 randomizations of rearing events (see text).

**Figure 4.**
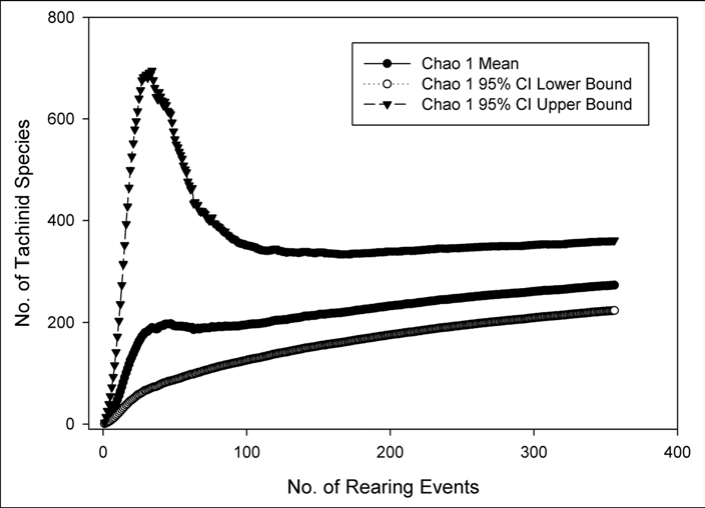
Mean Chao-1 estimates of total species richness of Tachinidae with sample size (number of rearing events) along with upper and lower 95% confidence limits. The mean estimate at full sample size is 273 species.

Both the empirically observed species accumulation curve and the estimated Mau Tau rarefaction curve suggest that we have sampled only a fraction of the diversity of Lepidoptera-using Tachinidae at YBS and surrounding areas (Figure 3). The observed species accumulation curve is nearly a straight line (with a slight plateau near the center representing a time period in which a large number of the same host caterpillar was reared), with no indication of asymptotic behavior. The shape of the rarefaction curve suggests that the rate of new species accumulation may be decreasing from its initial values due to multiple rearing events of some species, but the rate still remains high (approximately one new species for every four rearing events). The Chao-1 estimator of total species richness predicts that the community of Lepidoptera-attacking Tachinidae in the sampled area consists of approximately 273 species, with a 95% confidence interval between 224 and 361 species (Figure 4; Table 2). Although the estimates stabilized to some extent after approximately 100 rearing events, both the mean estimates and associated confidence intervals continue to increase at a relatively constant rate. Most of the other species richness estimators resulted in similar estimates of 270–300 species (Table 2), although Jack-1 and bootstrap estimates predicted somewhat lower total species richness.

**Figure 5.**
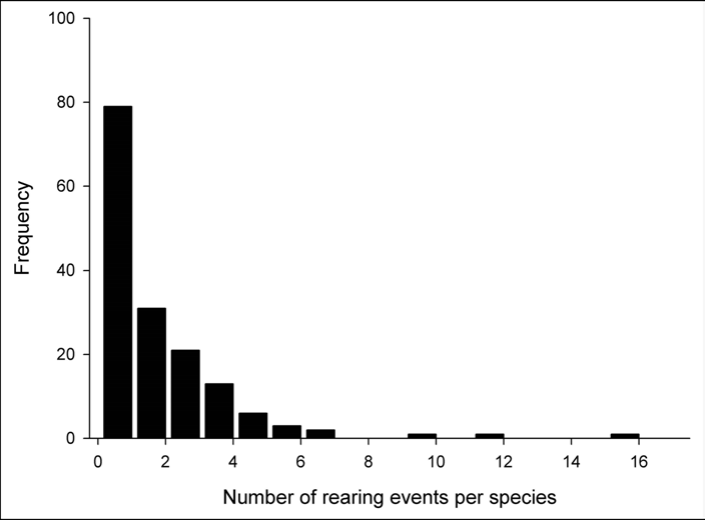
The species abundance distribution of tachinids reared in the current study in terms of number of rearing events per species.

**Figure 6.**
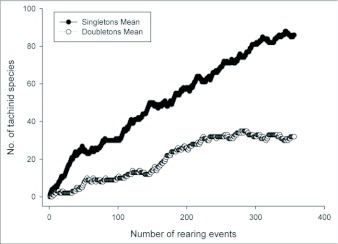
Observed accumulation of singletons (species recorded only once) and doubletons (species recorded twice) with increasing number of tachinid rearing events.

**Figure 7.**
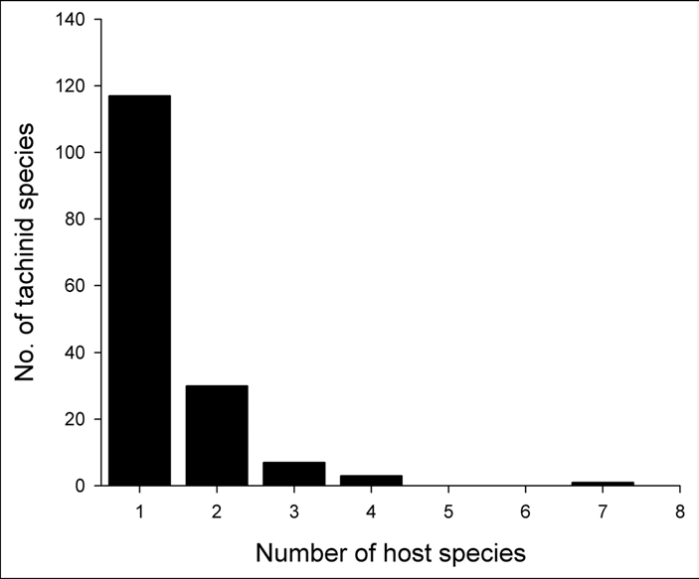
Frequency histogram of the number of host species from which each tachinid species was reared.

**Figure 8.**
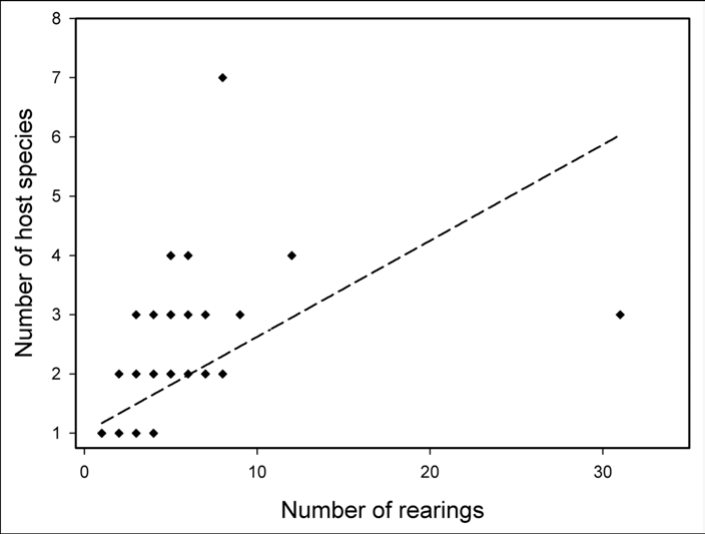
Least squares regression of the number of host species against the number of rearing events. (regression equation: y = 0.273x + 0.76, R^2^ = 0.354, p < 0.0001).

One half of the tachinid “species” reared (79 spp.), were reared only once (i.e., singletons; although in many cases these were gregarious and are represented by multiple individuals; Figure 5; Table 1). Another 20% (31 spp.) were reared on two occasions (doubletons), and only about 2% of species were reared ten or more times, resulting in a highly left-skewed abundance distribution that best fits a log-series distribution (p = 0.9998; (Figure 5). The number of singletons increases nearly linearly with rearing events with no clear evidence of leveling off, suggesting that many rare species remain to be sampled and that sampling intensity, at this point, is inadequate to characterize the community well ((Figure 6). In contrast, numbers of doubletons, are beginning to stabilize and even decline slightly, perhaps due to recent attempts to widen the diversity of host caterpillars sampled. Most of the tachinid species (74%) were reared from only a single host species, and no species was reared from more than seven host species (Figure 7). The mean number of host species per tachinid species was 1.38 ± 0.06 (SE) and the mean number of host families was 1.16 ± 0.04. However, these figures reveal little about actual host ranges, as most tachinids were only reared a single time (see above), and therefore could not have been reared from multiple host species. A significant relationship exists (R^2^ = 0.354, P < 0.0001; Figure 8) between the inferred host range of tachinid species and the number of rearing events, as has been found in other analyses of tachinid host ranges (e.g., Eggleton and Gaston 1992; Belshaw 1994). This result suggests that the perceived host ranges of the Ecuadorian tachinid species are likely to increase with greater sampling effort. General host associations of tachinid genera obtained from the rearing data are listed in Table 1.

**Table 1.**
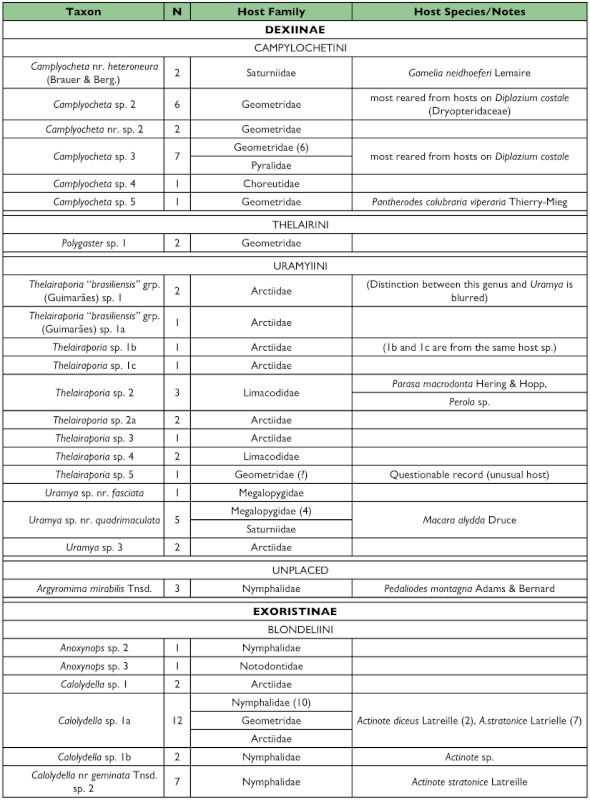
list of the tachinid genera thus far reared from caterpillars from the Yanayacu Biological Station and surrounding areas. The number of rearing events (N), the host families from which they have been reared, and notes about particular taxa are indicated.

**con't:**
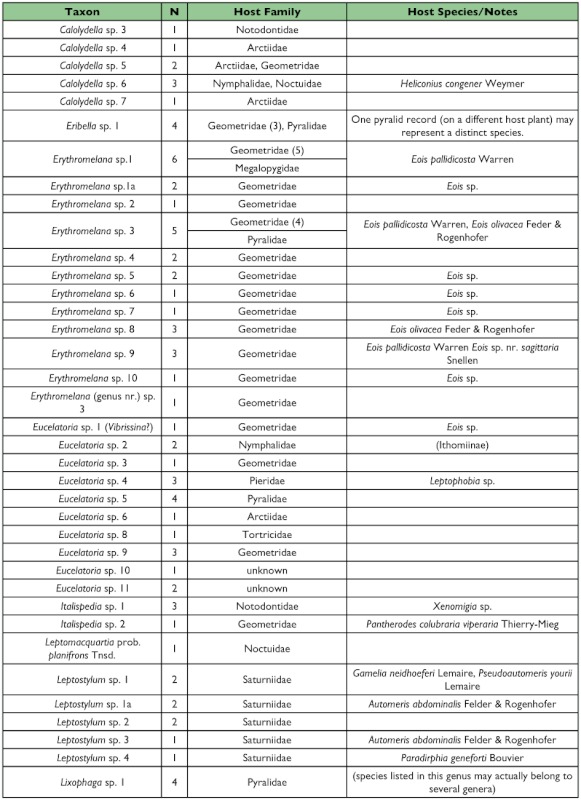

**con't:**
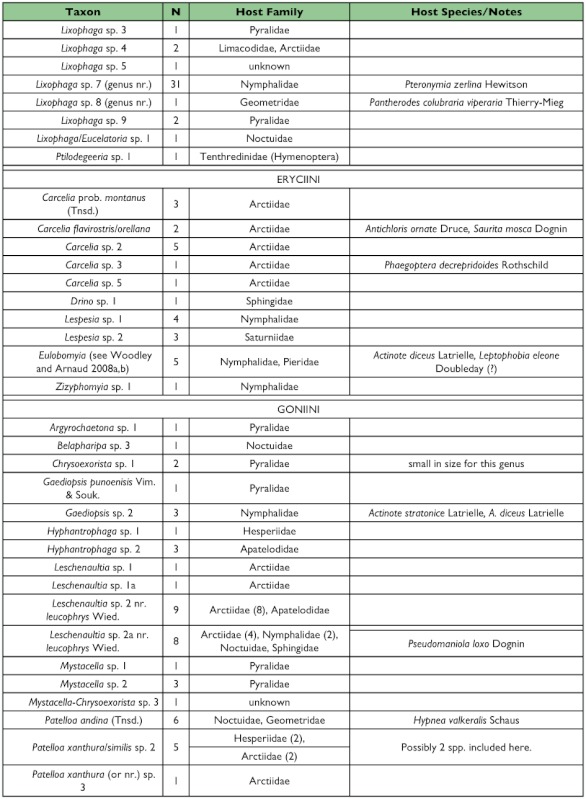

**con't:**
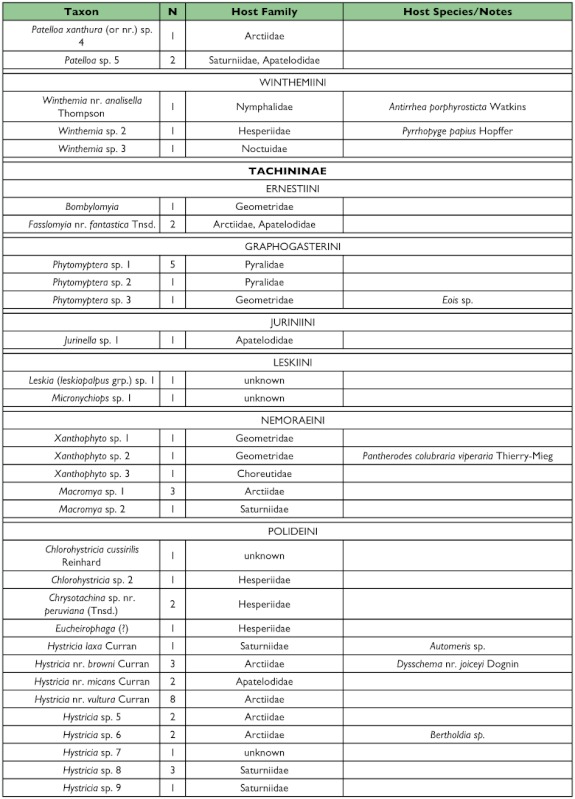

**con't:**
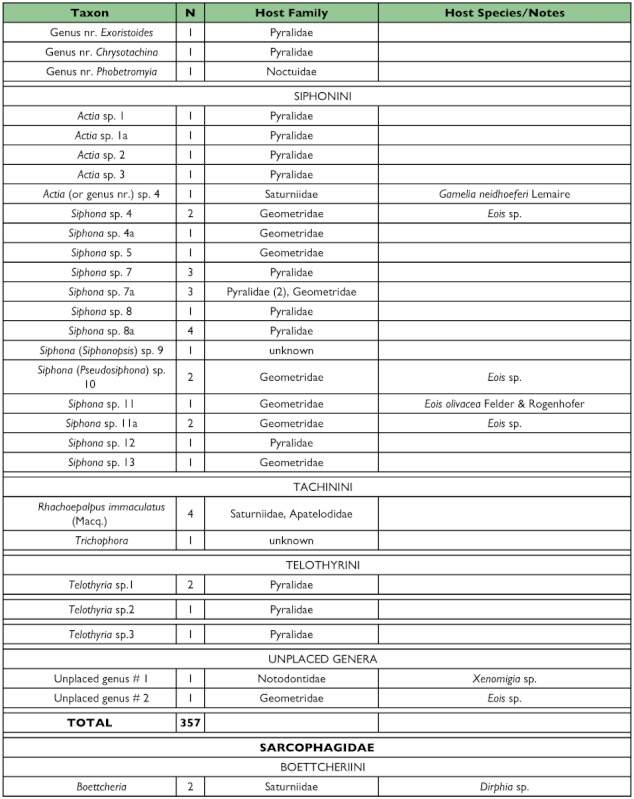

**Table 2.**
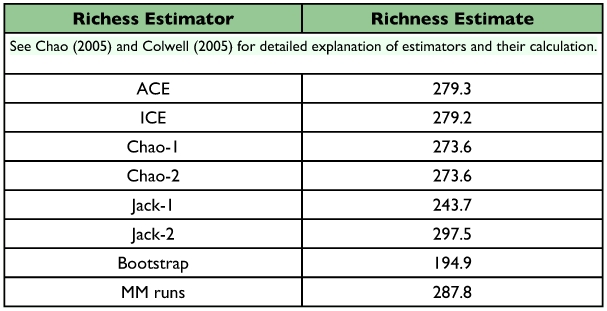
Mean estimates of total species richness of the tachinid community of Yanayacu Biological Station and surrounding areas that use Lepidoptera as hosts, based on 1000 randomizations.

## Discussion

### Species Richness

Given the linear trajectory of the species accumulation curve and the only slightly declining slope of the rarefaction curve, it is difficult to gauge how many species of Tachinidae might occur in the sampled region. Furthermore, there are few equivalent studies of Tachinidae with which to compare these estimates. In a previous Lepidoptera rearing study in southern Arizona, Stireman and Singer (2003b) reared 64 tachinid species in approximately 1240 rearing events. That study sampled from a considerably larger area than does the current one, but included fewer individuals and species of caterpillar hosts. Nonetheless, rarefaction indicates that an equivalent sampling effort in Arizona, in terms of rearing events (357), would yield a considerably smaller figure of approximately 46 tachinid species (JOS, unpub data), indicating a much larger fauna at the Ecuador site. Recent Caterpillar rearing studies in the eastern United States have yielded fewer tachinid species (e.g., ca. 30 by Barbosa and Caldas [2004], and 24 by Stazanac et al. [2001]), but these studies were narrower in scope, limiting the usefulness of comparison.

A recent catalogue places the number of Nearctic tachinids (north of Mexico) at 1345 species (O'Hara and Wood 2004). Assuming that 75% of these species use Lepidoptera as hosts, an estimated 1076 species could be reared from Lepidoptera. This suggests that the relatively small area sampled in and around YBS (ca. 200 km^2^) in Ecuador is home to about a quarter as many tachinid species as the entire Nearctic Region north of Mexico (ca. 20 million km^2^). This difference in the areas sampled is conservative, as most of the samples at YBS were taken from ca. 10 km^2^ surrounding the station. Furthermore, the estimators of total numbers of species at YBS (e.g., ICE, Chao-2) all exhibit signs of continuing increase with more rearing events (e.g., Figure 4). These estimators may not, therefore, be reliable estimates of total richness, but instead represent lower bounds (Longino et al. 2002).

The high diversity of tachinids in this mid-elevation tropical Andean site is also indicated by preliminary examination of tachinid specimens (ca. 1000 individuals) obtained by hand collecting around the YBS and in surrounding areas, which suggest relatively little overlap with reared material (unpub. data). This lack of overlap persists even when considering only those groups that are likely to use Lepidoptera as hosts (e.g. Goniini, Tachinini, Eryciini), supporting the prediction that many additional species remain to be reared from Lepidoptera in this area. The species abundance distribution (Figure 5) suggests a preponderance of rare species (70% singletons and doubletons). However, as this distribution reflects in large part inadequate sampling, it is premature to make conclusions concerning the relative abundance and rarity of species or higher taxa.

It has been suggested that species richness of some hymenopteran parasitoid groups may not increase with decreasing latitude or may increase more slowly than other insect taxa (Janzen and Pond 1975; Janzen 1981; though see Sääksjärvi et al. 2004). This trend may be due to greater resource fragmentation and/or increasingly toxic chemical defenses in potential hosts in the tropics (e.g. Janzen 1981; Gauld et al. 1992). The high species richness of Tachinidae at this site relative to similar Lepidoptera-rearing studies in more temperate areas (e.g., Stireman and Singer 2003b) suggests that tachinids exhibit a marked latitudinal gradient in species richness similar to their (primarily) phytophagous insect hosts. One speculative reason for this difference is that tachinids may be relatively less affected by resource fragmentation and host chemical defenses.

### Accuracy of species delimitations

As discussed in the Methods, there are several factors which may have resulted in an overestimation of tachinid diversity in the current study. For example, extensive intraspecific variation or sex-specific morphological variation could lead to splitting of one species into multiple morpho-species. These problems are somewhat mitigated by the approach employed here of rearing hosts to collect parasitoids, where multiple individuals (often both sexes) are frequently reared from a single host. In addition, associations of parasitoids with particular host taxa can provide ecological evidence that may support morphological divisions of the parasitoids, as exemplified in a recent study of the tachinid genus *Belvosia* in Costa Rica (Smith et al. 2006). However, if understanding patterns of host association and host range is a goal, using this information in defining parasitoid species may result in tautological reasoning.

It is also possible that the number of distinct morpho-species identified here is actually an underestimate of the true species diversity. Observed variation among species may be easily ascribed to intra-specific variation and considerable existing variation may go unrecognized. Given recent evidence of sympatric, morphologically cryptic, but apparently reproductively isolated populations of tropical tachinids that differ in host use (Smith et al. 2006; Smith et al. 2007), it seems likely that the species richness reported here is underestimated. The great diversity and confusing taxonomy of Neotropical tachinids suggests that the region may be an “epicenter” of recent tachinid diversification (Stireman et al. 2006; supporting the general pattern of elevated speciation rates in the tropics; Mittelbach et al. 2007). Furthermore, tachinid diversity appears to peak at intermediate elevations in the tropics (Wood, pers. comm.). Thus, areas such as YBS, at middle-elevations on the eastern equatorial Andes, may be particularly rich with morphologically similar (i.e., cryptic) tachinid species.

It should be noted that currently fewer than 10% of the reared species have been assigned to a named species (or “near” a named species), and it could be conservatively estimated that perhaps 75% or more have not been described. Similarly, it is probable that >90% of the species have previously never been associated with a host in the scientific literature. This figure may be revised downward as we gain a better understanding of the overlap in taxa between this Ecuadorian site and other major Lepidoptera rearing efforts in Costa Rica (Gentry and Dyer 2002; Janzen and Hallwachs 2007). Many of the tachinids reared in this study are currently featured in a preliminary online guide to the tachinids of Ecuador with photos, taxonomic notes, and life history information (see http://www.caterpillars.org).

### Distribution of Taxa

Certain subfamilies and tribes of Tachinidae were extremely well represented in the collection of reared species and others poorly so (Table 1). In part, this is due to the focus on lepidopteran hosts, such that higher taxa containing species that primarily attack non-lepidopteran hosts such as Phasiinae and Dexiini are not well represented. However, relative frequencies of taxa reared from caterpillars are generally consistent with observed frequencies of taxa in hand-collected samples from the same area. For example, extensive hand collections made along roadsides and trailsides contained no representatives of Phasiinae. Dexiini were also rare in the hand collections, despite extensive collecting effort on flowers (e.g., Asteraceae) where many dexiine genera (and Phasiinae) often take nectar and/or pollen in other regions. Voriini were also markedly absent in collections obtained by hand and through host rearing, despite the general use of Lepidoptera as hosts by members of this tribe (e.g., Geometridae; Arnaud 1978).

Over one half of all species reared (and one half of all tachinid rearing events) belong to the subfamily Exoristinae. Blondeliini was the most frequently reared tribe and was represented by a diverse assemblage of closely related genera, most of which were represented by several species (e.g., *Calolydella, Eucelatoria, Lixophaga, Erythromelana*). This tribe accounted for over 30% of both rearing events and tachinid species reared. Goniini (Figure 1a, 1d) and Eryciini were also responsible for appreciable numbers of parasitism events (12.6% and 5.7% of species reared respectively), although certain taxa well represented in D.H. Janzen and W. Hallwach's (2007) caterpillar rearing database, such as *Drino, Lespesia*, and *Belvosia* were noticeably rare or absent. The genus *Winthemia*, which is commonly reared from macrolepidoptera in the temperate zone and Central American tropics (Arnaud 1978; Janzen and Hallwachs 2007), was also conspicuously underrepresented.

The subfamily Tachininae was well represented (33.5% of reared species), but in this case there was incongruence between reared and hand-collected taxa. Field observations and hand-netting indicate an inordinately diverse fauna of Tachinini, particularly the “big fuzzy” taxa such as *Epalpus* (Figure 1c) and allied genera (e.g., *Lindigepalpus, Parepalpus, Eulasiopalpus*). Despite these observations, few Tachinini were reared (only 1.3% of total species). This may be due to the relatively low rearing numbers of large-bodied caterpillar taxa, such as Sphingidae and Saturniidae, capable of hosting these bulky tachinids. These large and active tachinids may also be relatively more apparent in the field than other tachinid taxa (i.e., their abundance is overestimated due to their conspicuousness). In contrast, Siphonini were common and diverse in both reared (11.4% of species) and netted collections. The relatively large number of polideine species reared (15 species) was somewhat surprising, given the limited representation of this tribe in other Lepidoptera rearing studies (e.g., Stireman and Singer 2003b). Much of this diversity of Polideini was a function of the large number of species (9) of the tachinine-like genus *Hystricia*.

### Host associations

Most of the family-level host associations of taxa reported here (Table 1), are consistent with previously established host-associations of particular tachinid groups (Guimarães 1977; Arnaud 1978). For example, *Carcelia* and *Leschenaultia* (Figure 1d) species were associated primarily with hosts in the family Arctiidae; uramyines were associated with Megalopygidae, Arctiidae, and Limacodidae; *Eucelatoria* species were primarily associated with Geometridae; and siphonines were associated with hosts of small size such as Geometridae and Pyralidae. Additional strong associations that were observed included *Leptostylum* with Saturniidae (Figure 1b) and *Erythromelana* with Geometridae. It is interesting to note that many *Lixophaga* spp. were reared from shelter-building Pyralidae larvae, reflecting the ability of their host searching larvae to attack of concealed hosts.

Although a thorough evaluation of the parasitism frequency of various ecological guilds or phylogenetic clades of hosts is beyond the scope of this paper, a preliminary examination of parasitism frequency by host family suggests that tachinids do not use all hosts with equal frequency. For example, the families Limacodidae, Megalopygidae, Sphinigidae, Saturniidae, and Arctiidae are attacked at relatively high frequencies by tachinids (<10%), while Geometridae, Noctuidae, and Choreutidae were parasitized less frequently. This variation probably reflects host ecology more than phylogenetic or physiological interactions between host and parasitoid (Stireman and Singer 2003a). Interestingly, despite their shelter building habit and small size, Pyralidae experienced relatively high parasitism by tachinids, possibly due to the attraction of parasitoids by the copious frass built up inside shelters (Roth et al. 1978).

It has been suggested that the host associations of tachinid species may depend more upon the microhabitat and diet of potential host species than the taxonomic/phylogenetic affiliations of the hosts (Crosskey 1980; Feener & Brown 1997; Stireman and Singer 2003a). This may reflect the importance of host location mechanisms in shaping patterns of host use and the indirect strategies by which many tachinids parasitize hosts (Feener & Brown 1997; Stireman et al. 2006). Although the current study provides several clear examples of broad associations between tachinid and host taxa (see above), many associations also support an important role of host-plants in shaping patterns of tachinid host use. For example, *Uramya* nr. *quadrimaculata* was reared primarily from Megalopygidae (one of the typical host families for this tachinid genus; Arnaud 1978); however, this species was also reared from a completely unrelated species of Saturniidae that fed on the same host plant (*Acalypha*, sp.; Euphorbiaceae). As another example, *Erythromelana* species tended to be associated with Geometridae, especially *Eois* sp. on plants in the genus *Piper*, but at least one species was also found to attack unrelated Pyralidae on the same *Piper* host plants.

Due to the relatively low sampling intensity and infrequent rearing events of particular tachinid species, little can be surmised concerning the breadth of host use for the tachinid species reared in this study. Observed host associations suggest, however, that there are few, if any, true generalists in this fauna and that most species are likely specialized on a few taxonomically or ecologically related host species. This contrasts with some analyses of host range in temperate tachinid taxa (Eggleton and Gaston 1992; but see Stireman and Singer 2003b), suggesting that there may exist a latitudinal gradient in specialization in Tachinidae as has been indicated for their lepidopteran hosts (Dyer et al. 2007).

### Future Directions

Considerably more host rearing will be necessary to achieve a robust understanding of the diversity and taxonomic composition of this local community of Tachinidae. Despite the relatively large number of species reared thus far, it is clear that only a fraction of tachinid species in this diverse community have been sampled. Focused collection and rearing of caterpillars from the forest canopy may be one strategy to obtain greater proportion of the community of tachinid species that attack Lepidoptera. Furthermore, rearing of additional herbivorous insect taxa such as Hemiptera, Symphyta, and Chrysomelidae would significantly expand the number of species and taxonomic diversity of tachinids recorded from the region. The caterpillar rearing program described here is currently being augmented by hand-netting as well as pan-trapping and Malaise trapping, which will allow more exhaustive estimation of the total richness of the tachinid community, as well as a more thorough examination of biases associated with different sampling methods.

In addition to establishing the patterns of diversity and host associations of Tachinidae, much of the material reared from this project will contribute to future species descriptions, taxonomic revisions, and phylogenetic analyses of this poorly known Neotropical fauna. One benefit of the rearing approach to sampling employed here is that it provides basic natural history and life history information such as host-associations, plant-associations and developmental phenology. In addition to the inherent worth of this information, it may prove useful in establishing species limits and phylogenetic relationships. Another benefit of the approach is that in gregarious species (i.e., those in which multiple individuals can develop in a single host) sexes can be associated. This limits the possibility of each sex being described as a distinct species. As recent studies of Costa Rican tachinids have shown (Smith et al. 2006; 2007), DNA data can aid substantially in delineating species. DNA samples are now being gathered for mtDNA “barcode” sequencing of the reared tachinids, which may also be useful comparing this Andean tachinid fauna to that in Costa Rica to assess species turnover and geographic population structure of species.

Our current lack of understanding of tropical biodiversity is particularly alarming when one considers the rate of tropical deforestations and estimated rates of species extinctions measured on the scale of species loss per week or month (Pimm and Raven 2000). Given their specialized life histories and elevated trophic position, tropical parasitoids may be particularly prone to extinction via habitat loss. This problem is compounded due to a current taxonomic crisis (Brown 2005), wherein the rate of description of new species from the Neotropical Region is extremely low, suggesting that many species are quietly disappearing without ever being recorded. A major goal of this study and associated research is to provide baseline documentation of the diversity and natural history of caterpillars and parasitoids in the Ecuadorian Andes. The results reported here, on the richness and host associations of Tachinidae, represent a small but important step towards understanding tropical insect diversity.

### Editor's note

Paper copies of this article will be deposited in the following libraries. Senckenberg Library, Frankfurt Germany; National Museum of Natural History, Paris, France; Field Museum of Natural History, Chicago, Illinois USA; the University of Wisconsin, Madison, USA; the University of Arizona, Tucson, Arizona USA; Smithsonian Institution Libraries, Washington D.C. USA; The Linnean Society, London, England.
